# Discovery of new small molecules inhibiting 67 kDa laminin receptor interaction with laminin and cancer cell invasion

**DOI:** 10.18632/oncotarget.4016

**Published:** 2015-05-29

**Authors:** Ada Pesapane, Carmen Di Giovanni, Francesca Wanda Rossi, Daniela Alfano, Luigi Formisano, Pia Ragno, Carmine Selleri, Nunzia Montuori, Antonio Lavecchia

**Affiliations:** ^1^ Department of Translational Medical Sciences, University of Naples Federico II, Naples, Italy; ^2^ Department of Pharmacy, Drug Discovery Laboratory, University of Naples Federico II, Naples, Italy; ^3^ Institute of Genetics and Biophysics Adriano Buzzati-Traverso, Consiglio Nazionale delle Ricerche (CNR), Naples, Italy; ^4^ Department of Science and Technology, University of Sannio, Benevento, Italy; ^5^ Department of Chemistry, University of Salerno, Salerno, Italy; ^6^ Department of Medicine and Surgery, University of Salerno, Salerno, Italy

**Keywords:** laminin receptor, small molecules, laminin, cell adhesion

## Abstract

The 67 kDa laminin receptor (67LR) is a non-integrin receptor for laminin (LM) that derives from a 37 kDa precursor (37LRP). 67LR expression is increased in neoplastic cells and correlates with an enhanced invasive and metastatic potential.

We used structure-based virtual screening (SB-VS) to search for 67LR inhibitory small molecules, by focusing on a 37LRP sequence, the peptide G, able to specifically bind LM. Forty-six compounds were identified and tested on HEK-293 cells transfected with 37LRP/67LR (LR-293 cells). One compound, NSC47924, selectively inhibited LR-293 cell adhesion to LM with IC_50_ and K_i_ values of 19.35 and 2.45 μmol/L.

NSC47924 engaged residues W176 and L173 of peptide G, critical for specific LM binding. Indeed, NSC47924 inhibited *in vitro* binding of recombinant 37LRP to both LM and its YIGSR fragment. NSC47924 also impaired LR-293 cell migration to LM and cell invasion.

A subsequent hierarchical similarity search with NSC47924 led to the identification of additional four compounds inhibiting LR-293 cell binding to LM: NSC47923, NSC48478, NSC48861, and NSC48869, with IC_50_ values of 1.99, 1.76, 3.4, and 4.0 μmol/L, respectively, and able to block *in vitro* cancer cell invasion.

These compounds are promising scaffolds for future drug design and discovery efforts in cancer progression.

## INTRODUCTION

The 67 kDa laminin receptor (67LR) was originally identified as a non-integrin cell surface receptor for LM [1], the major component of basement membranes [2]. Interactions between 67LR and LM play a major role in mediating cell adhesion [3], migration [4], proliferation and survival [5].

67LR derives from homo- [6] or hetero- [7] dimerization of a 37 kDa cytosolic precursor (37LRP) [8], most probably by fatty acid acylation. 37LRP is mostly found in the cytosol [9] and nucleus [10] where it is involved in translational processes and maintenance of nuclear structures, respectively. 67LR is localized at the cell surface [11] and it not only serves as a receptor for LM but also acts as a receptor for elastin [12], carbohydrates [13] and the cellular prion protein [14].

67LR binds LM through different binding domains: a palindromic sequence known as peptide G [11, 15], a predicted helical domain corresponding to 37LRP residues 205–229, and TEDWS-containing C-terminal repeats [13]. 67LR is co-expressed and can physically interact with the α_6_ integrin chain [16]. LM conformation changes upon binding 67LR, thus interacting more efficiently with integrins [17] and becoming more sensitive to the action of proteolytic enzymes [18], with the release of motility fragments [19].

67LR expression is increased in neoplastic cells as compared to their normal counterparts and directly correlates with an enhanced invasive and metastatic potential [20], mediated by high-affinity interactions between 67LR and LM [21].

Cell adhesion to the basement membrane allows tumor cells to secrete proteolytic enzymes, i.e. type IV collagenase, able to degrade components of the extracellular matrix (ECM). Degradation of these components, in turn, induces invasion of the basement membrane, enabling cancerous cells to migrate and form metastasis. Thus, 67LR overexpression is a molecular marker of aggressiveness in cancers of many tissues, including breast, lung, ovary, prostate and also in leukaemia and lymphomas [22–24].

The correlation between 37LRP/67LR levels and tumor aggressiveness recommends the receptor as a new promising target for cancer treatment. This is supported by *in vivo* studies showing that high 67LR levels result in tumor growth and proliferation [25, 26]. Knockdown of 37LRP using siRNAs resulted in decreased cell survival suggesting that 37LRP/67LR could also enhance cell viability by blocking apoptosis [27]. Indeed, we recently demonstrated the structural and functional association of 67LR with the anti-apoptotic protein PED/PEA-15 [28]. Furthermore, recent findings demonstrated that an anti-37LRP/67LR specific antibody significantly impeded angiogenesis, thus suggesting the receptor might also be involved in tumor angiogenesis [29].

However, an anti-37LRP/67LR specific antibody decreased the invasive potential of human fibrosarcoma cells [30], thus indicating that 67LR plays a critical role in tumor invasion and metastasis through its interaction with LM.

The 2.15 Å resolution crystal structure of the partial domain of human 67LR [31] provides an excellent platform for rational drug design. For these reasons, we used structure-based virtual screening (SB-VS) [32] of the National Cancer Institute (NCI) Diversity Set with nonredundant structures to identify small molecules targeting 67LR and able to disrupt cell binding to LM. SB-VS, which uses computer-based methods for identifying promising compounds to bind to a target molecule of known structure, is a widely used method that has been shown to be successful in a variety of studies, although it also has many shortcomings [33].

Here, we describe the successful outcome of this search and the initial biological evaluation of the most promising compounds from this effort.

## RESULTS

### Identification of a druggable pocket within the human 67LR structure

**Figure 1:**
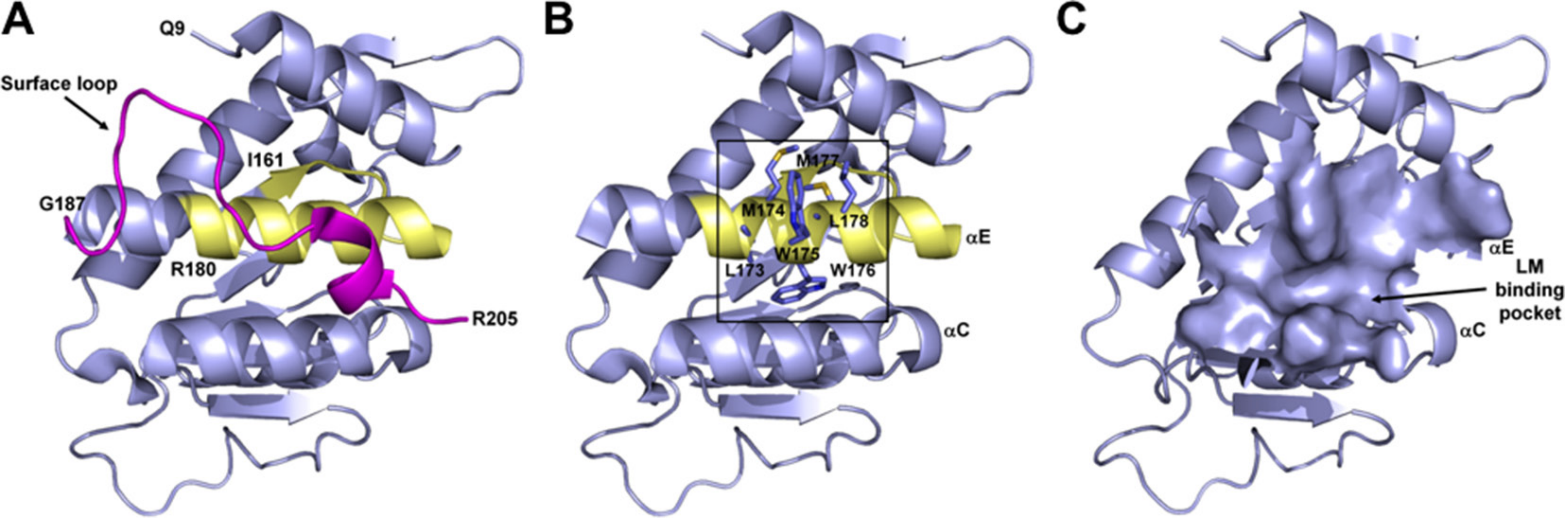
Structure of human 37LRP **A.** Structure of human 37LRP represented as a slate blue cartoon. The structure begins at residue Q9 and finishes at residue R205, lacking almost all the C-terminal LM-binding site, which starts at residue R205. Peptide G (residues 161–180) is shown in yellow. The surface loop (residues 187–205), which covers the palindromic sequence of peptide G, is shown in magenta. **B.** Structure of human 37LRP lacking of the surface loop. The palindromic sequence of peptide G (LMWWML) is circled in black. **C.** The 2265 Å^2^ putative LM binding pocket lying between αC and αE helices of 37LRP. Figures were produced using the coordinates from Protein Data Bank file 3BCH (35) and PyMOL (www.pymol.org).

Recently, the structure of the N-terminal of 37LRP (residues 1–220) has been solved by X-ray crystallography [31] with resolution of 2.15 Å (Protein Data Bank ID code 3BCH) (Figure 1A). 37LRP was shown to have a globular structure comprising five α-helical and seven β-folded regions. This structure shows a high degree of similarity to ribosomal protein SA or p40 from prokaryotes and lower eukaryotes [9, 34]. Since 37LRP crystal structure begins at residue 9 (Q9; single-letter amino-acid code) and finishes at residue 205 (R205) (both indicated on the Figure 1A), it lacks almost all the C-terminal domain, not present in the prokaryotic and lower eukaryotic ribosomal proteins [35], which starts at residue 205.

Among the different 67LR binding sites for LM, we focused on peptide G for the abundant clinical and experimental data indicating its critical role in tumor invasion and metastasis [11, 15, 17–21]. Peptide G (residues 161–180, IPCNNKGAHSVGLMWWMLAR) binds LM with high affinity (K_d_ = 51.8 nM) [11, 15, 17]. Moreover, evolutionary studies suggested that the acquisition of the LM-binding capability of 67LR is linked to the palindromic sequence LMWWML contained within the peptide G [35]. Peptide G forms part of a β-strand (residues 160–162), an α-helix (residues 168–186) and a surface loop (residues 187–205), much of which is buried in the interior of the molecule. The only portion of it that is solvent-accessible includes residues 165–169.

The crystal structure of 37LRP reveals that the surface loop completely covers the palindromic sequence of peptide G, making it inaccessible to binding of LM (Figure 1A); thus, it has been postulated that considerable conformational changes are required to enable LM binding. Indeed, we found that loop 188–197 in the C-terminal region is highly flexible and undergoes a major change resulting in a conformational switch that partially solvent exposes the final part of peptide G [36].

Thus, with the aim of exposing the palindromic sequence (Figure 1B) involving protein-LM interactions, we truncated the surface loop of 37LRP (residues 187–205) from the available crystallographic structure. One cavity was intercepted in close proximity of the palindromic residue W176. This groove lies between two α helices (αC and αE) and covers a surface area of 2265 Å^2^ (Figure 1C). The site is predominantly hydrophobic in nature (L173, M174, M177, L178, A86, G172, A93, A179, G172, A168, F90, W175, W176) with a few H-bond donors and acceptors. This putative cavity, that we hypothesize to be involved in LM binding, was chosen for *in silico* targeting in a SB-VS approach.

### Identification by SB-VS of small molecules directed to the peptide G cavity of 67LR

An *in silico* screening approach was undertaken to identify compounds from the NCI Diversity library with a potential to bind at the putative cavity on the peptide G of 67LR and hamper LM binding. 3D structures of compounds from the NCI's chemical libraries were downloaded from the NCI Developmental Therapeutics Program web site (http://dtp.nci.nih.gov/branches/dscb/repo_open.html/) and processed with LigPrep software to produce 2, 560 3D structures for the Diversity Set. Docking calculations were carried out by using the Glide software [37]. Then, Glide SP docked each chemical structure into the 67LR/LM binding site retaining the 10% of the top-scoring ligands. The resulting 188 compounds were then redocked and scored with Glide XP in order to estimate binding affinity and rank the ligands. The 188 ranked compounds were analyzed by visual inspection because it has broadly demonstrated that docking scoring functions are often more successful at predicting a binding pose than the actual binding affinity [38]. Finally, compounds were checked for readily sample availability from the compound provider, and 46 structurally diverse compounds (compounds 1-46 in Chart 1 of Supplementary Material: SB-VS selected small molecules directed to the peptide G cavity of 67LR) were requested and tested in a cell-based assay.

### Human 37LRP cDNA transfection in HEK-293 cells results in cell surface expression of the active receptor and increased cell adhesion to LM

As a model to mimic the effects of 67LR overexpression in cancer cells, HEK-293 cells were stably transfected with a human 37LRP cDNA fused at the C-terminal with a tag derived from phage T7 and a poly-histidine stretch (LR-293 cells) or with an empty vector (V-293 cells).

**Figure 2:**
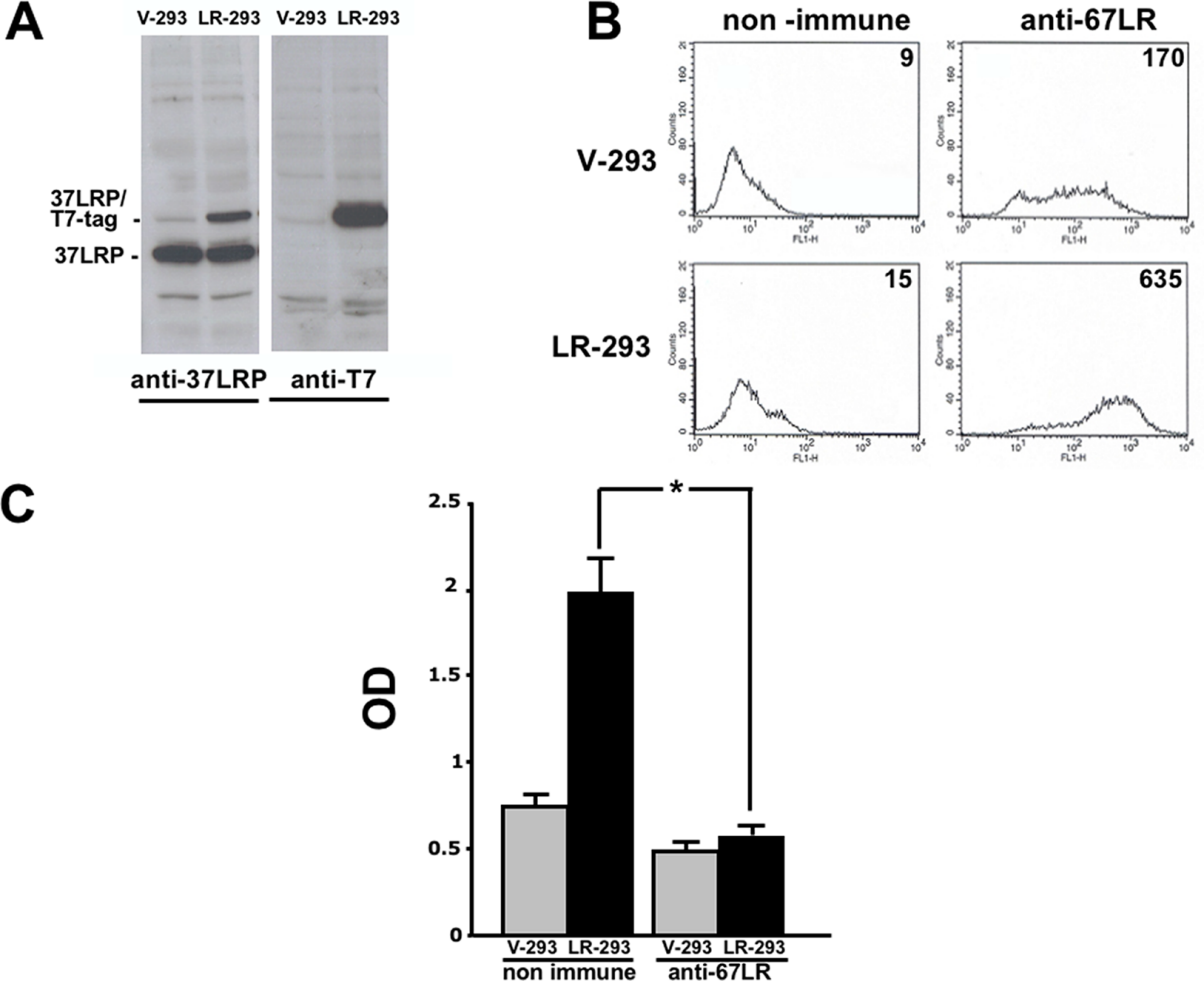
37LRP cDNA transfection in HEK-293 cells results in cell surface expression of the mature receptor and increased cell adhesion to LM **A.** HEK-293 cells were transfected with 37LRP cDNA fused at the C-terminal with a T7 tag and a poly-histidine stretch (37LRP/T7-tag), and named LR-293, or with the empty vector, V-293. Transfected cells were lysed and 50 μg of protein was analyzed by Western blot with 37LRP/67LR and T7-tag specific antibodies. **B.** Flow cytometric analysis of cell surface 67LR expression was evaluated by incubating V-293 and LR-293 cells with a polyclonal anti-67LR antibody or an isotype control. Fluorescence intensity values are reported. **C.** V-293 (□) and LR-293 (■) cells were plated on LM-coated wells in the presence of 20 μg/ml non immune immunoglobulins or anti-37LRP/67LR polyclonal antibodies. The attached cells were stained and the absorbance at 540 nm was measured. The values represent the mean ± SD of three experiments performed in triplicate. (*) *p* < 0.05, as determined by the Student's *t* test.

37LRP expression was then evaluated by Western blot analysis, using an anti-37LRP/67LR polyclonal antibody and an anti-T7 tag monoclonal antibody, in V-293 and LR-293 cell lysates. LR-293 cells expressed both endogenous and transfected 37LRP (37LRP/T7tag), whereas V-293 cells expressed only endogenous 37LRP (Figure 2A).

To verify that transfected 37LRP was correctly processed into the mature form and over-expressed at the cell surface, flow cytometry analysis was performed on V-293 and LR-293 cells with the same polyclonal antibody. LR-293 cells showed increased 67LR surface expression in respect to V-293 cells (Figure 2B).

Finally, 67LR overexpressed on LR-293 cell surface was functionally active; indeed, LR-293 cell adhesion to LM was significantly increased, as compared to V-293 cells, and such an increase was completely abrogated by cell pre-treatment with anti-37LRP/67LR polyclonal antibodies (Figure 2C).

### Cell adhesion to LM of 67LR overexpressing cells is inhibited by SB-VS selected compounds

To identify potential molecules able to inhibit 67LR binding to LM, LR-293 cells were subjected to adhesion experiments on LM-coated wells in the presence of the 46 initial hits, dissolved in DMSO and added at a concentration of 2×10^−5^ M; DMSO alone was used as a negative control.

**Figure 3:**
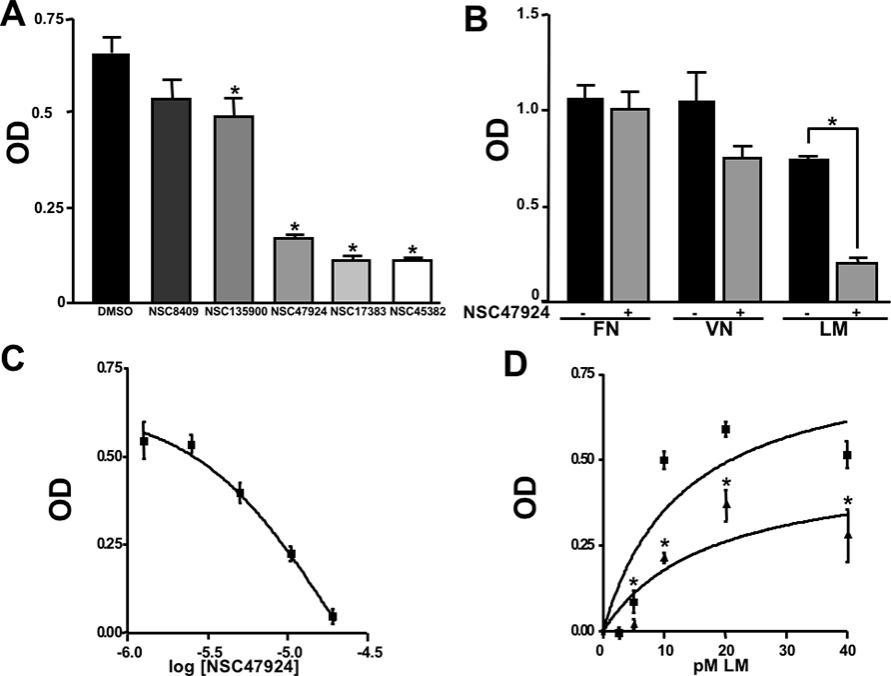
Five top-scoring molecules from SB-VS of a diversity library of small molecules inhibit LR-293 cell adhesion to LM; but, only one compound, NSC47924, shows specificity for LM **A.** LR-293 cells were plated in LM-coated wells in the presence of SB-VS selected molecules, at a concentration of 20 μM, DMSO was used as negative control (■). The attached cells were stained and the absorbance at 540 nm was measured. The values represent the mean ± SD of three experiments performed in triplicate. (*) *p* < 0.05, as determined by the Student's *t* test. Among the 46 small molecules tested, only five compounds were able to inhibit LR-293 cell binding to LM and are shown. **B.** LR-293 cell adhesion to LM, fibronectin (FN) and vitronectin (VN), in the presence of 20 μM NSC47924 (□) or DMSO, as a negative control (■). The attached cells were stained and the absorbance at 540 nm was measured. The values represent the mean ± SD of three experiments performed in triplicate. (*) *p* < 0.05, as determined by the Student's *t* test. NSC47924, specifically decreased LR-293 cell binding to LM, without affecting cell adherence to FN and VN. **C.** LR-293 cells were plated on LM-coated wells in the presence of decreasing concentrations of NSC47924. The attached cells were stained and the absorbance at 540 nm was measured. The values represent the mean ± SD of three experiments performed in triplicate. NSC47924 inhibits LR-293 cell adhesion to LM in a dose dependent manner. **D.** LR-293 cells were plated for 1 hour on wells coated with increasing concentrations of LM in the presence of NSC47924 (▲), at its IC_50_, or DMSO (■), as a vehicle control; attached cells were stained and the absorbance at 540 nm was measured. Values represent the mean ± SD of three experiments carried out in triplicate. The addition of NSC47924, at its Ic_50_, resulted in a significant loss of LR-293 cell binding to LM.

Among the 46 candidates, only five compounds (Table 1) were able to inhibit LR-293 cell adhesion to LM (Figure 3A). Moreover, all these compounds satisfy the druglikeness rules defined by Qikprop (QikProp, version 4.1, Schrödinger, LLC).

**Table 1:** Experimentally Determined % Cell adhesion Inhibition, XP Docking Scores, and Properties Predicted by QikProp from 46 Screened Compounds

Cpd	Structure	NCI number	MW^a^	QP log *P*^b^	QPlog *S*^c^	QP *P*_Caco_^d^	QP log Khsa^e^	Score (kcal/mol)	% inhibition
**13**	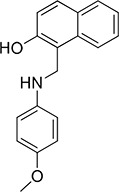	47924	279.3	4.0	–4.3	3784	0.365	–5.90	78
**20**	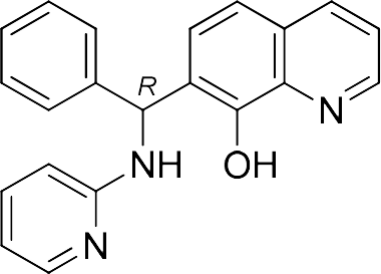	84094	327.4	4.4	–5.2	1686	0.572	–4.74	13
**30**	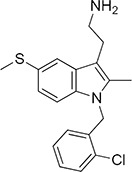	17383	344.9	5.0	–5.1	596	0.943	–3.82	93
**36**	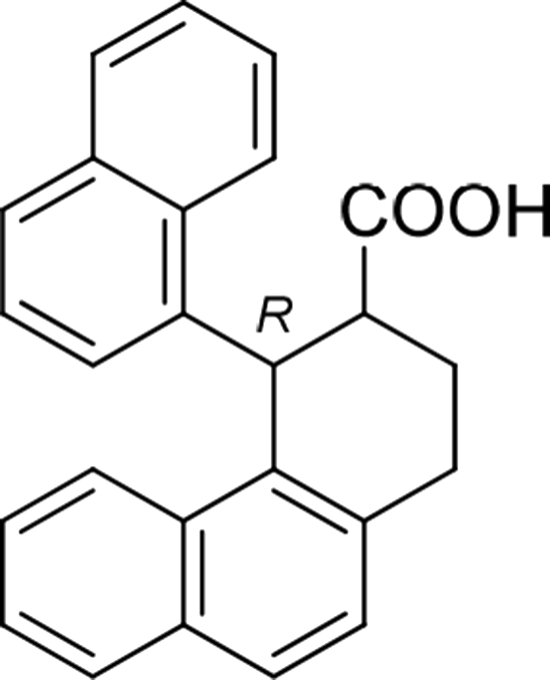	135900	352.4	5.7	–6.2	403	0.996	–4.89	37
**37**	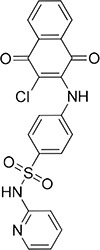	45382	439.9	2.0	–4.7	126	–0.225	–3.85	97

a MW, range 95% of drugs (130/725).

b Predicted octanol/water log *P*, range 95% of drugs (–2/6.5).

c Predicted aqueous solubility, *S*, in mol/L, range 95% of drugs (–6.5/0.5).

d Predicted Caco-2 cell permeability in nm/s, range 95% of drugs (<25 poor, >500 great).

e Predicted binding to human serum albumin, range 95% of drugs (–1.5/1.5).

### NSC47924 is a specific inhibitor of cell binding to LM

To verify their specificity, the five active compounds were tested for the ability to inhibit LR-293 cell adhesion to LM, FN and VN. Only one compound selectively inhibited LR-293 cell adhesion to LM. This molecule, NSC47924 [1-((4-methoxyanilino)methyl)-2-naphthol], strongly and specifically decreased cell binding to LM without significantly affect cell adherence to FN and VN (Figure 3B). On the contrary, the other compounds also inhibited cell binding to VN and FN (not shown) and were withdrawn from the study for their lack of specificity.

### NSC47924 inhibits cell binding to LM in a dose-dependent manner with a micromolar affinity

LR-293 cell adhesion to LM was also evaluated in the presence of decreasing concentration of NSC47924, in order to evaluate the dose-dependency of its inhibitory activity. IC_50_ value was 19.35 μM, as calculated by non-linear regression curves using the sigmoidal dose-response analysis of the GraphPad Prism software (Figure 3C). K_i_ of NSC47924 was 2.45 μM, as calculated by the Cheng and Prusoff Equation from EC_50_, using GraphPad Prism [39].

LR-293 cell adhesion to increasing concentrations of LM in the presence of NSC47924 at its IC_50_ was also evaluated. In the absence of inhibitors, LR-293 cell adhesion to LM showed a K_d_ of 14.5 nM, similar to that already reported for *in vitro* 67LR binding to LM [2 nM; ref. 40]; the addition of NSC47924, at its IC_50_, resulted in a significant loss of LR-293 cell binding to LM (Figure 3D).

### NSC47924 specifically inhibits 67LR-mediated cell adhesion, migration and invasion

67LR mediates high affinity interaction between cells and LM, thus increasing cell migration and invasiveness in overexpressing cells [21, 22–24, 30]; therefore, it was investigated whether NSC47924 was able to specifically inhibit 67LR-mediated cell adhesion and migration to LM and basement membrane invasion in LR-293 cells, using V-293 cells as a control.

**Figure 4:**
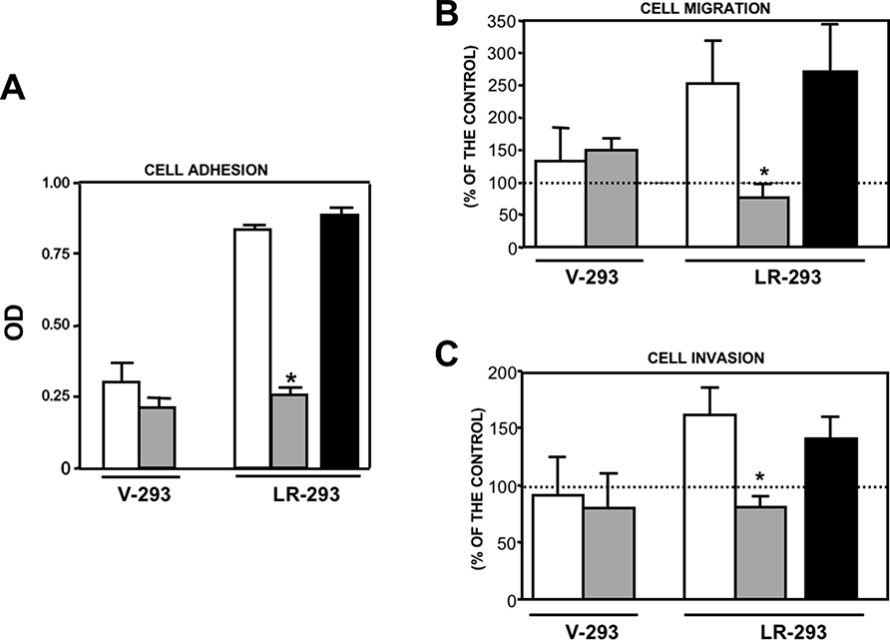
NSC47924 specifically inhibits 67LR-mediated cell adhesion and migration to LM as well as ECM invasion **A.** V-293 and LR-293 cells were plated on LM-coated wells in the presence of DMSO (□), as a vehicle control, 20 μM NSC47924 (□), or NSC373070 (■) as a negative control. The attached cells were stained and the absorbance at 540 nm was measured. The values represent the mean ± SD of three experiments performed in triplicate. (*) *p* < 0.05, as determined by the Student's *t* test. **B.** V-293 and LR-293 cells were pre-incubated with DMSO (□), 20 μM NSC47924 (□), or NSC373070 (■) as a negative control, plated in Boyden chambers and allowed to migrate toward 50 μg/ml LM on filters coated with 10 μg/ml FN. The values are the mean ± SD of three experiments performed in triplicate. (*) *p* < 0.05, as determined by the Student's *t* test. **C.** V-293 and LR-293 cells were preincubated with DMSO (□), 20 μM NSC47924 (□), or NSC373070 (■) as a negative control, plated in Boyden chambers and allowed to invade Matrigel^TM^. The values are the mean ± SD of three experiments performed in triplicate. (*) *p* < 0.05, as determined by the Student's *t* test. NSC47924 inhibited LR-293 cell adhesion and migration to LM as well as Matrigel invasion without exerting any significant effect on vector transfected V-293 cells.

To this aim, *in vitro* adhesion and migration assays to LM as well as Matrigel invasion assays were performed, in the presence or in the absence of 20 μM NSC47924. 67LR overexpressing LR-293 cells showed increased adhesion to LM, migration toward LM and Matrigel invasion, as compared to V-293 cells (Figure 4A, 4B, 4C). NSC47924 significantly inhibited LR-293 cell adhesion/migration to LM and invasion, as compared to vehicle treated cells; no significant effect was observed on V-293 cells (Figure 4A, 4B, 4C). NSC373070, an inactive compound derived from the 46 initial hits (Chart 1 of Supplementary Material), was added as a negative control and was ineffective in inhibiting LR-293 cell adhesion/migration to LM and Matrigel invasion, as expected (Figure 4A, 4B, 4C).

Thus, a novel small molecule specifically inhibiting cell binding to LM and its functional effects in 67LR overexpressing cells was identified by SB-VS.

### NSC47924 is a direct inhibitor of 37LRP/LM complex

To further demonstrate the specificity of 37LRP/67R as a target, the ability of NSC47924 to inhibit the binding to LM of human recombinant soluble 37LRP (r37LRP) was evaluated.

**Figure 5:**
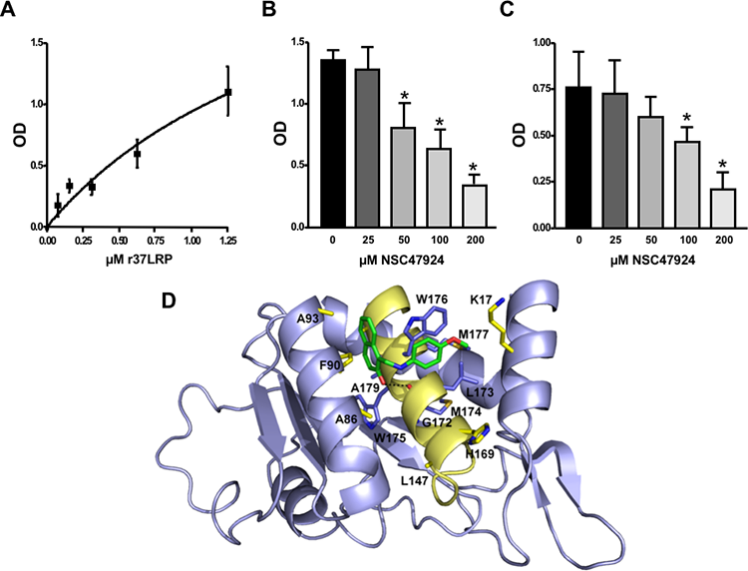
Structural basis of 67LR inhibition by NSC47924 **A.** Increasing concentrations of purified human His-tagged recombinant 37LRP (r37LRP) were placed for 1 hour at 37°C on wells coated with 1 μg of LM. Bound r37LRP was revealed by anti-His-HRP and OPD staining; the absorbance at 490 nm was measured. r37LRP binding to BSA-coated wells was subtracted to obtain specific binding. Values represent the mean ± SD of three experiments carried out in triplicate; (*, *P* < 0.05), as determined by the Student's *t* test. Human r37LRP binds to LM in a dose dependent manner. **B.** r37LRP was placed for 1 hour at 37°C on LM-coated wells in the presence of increasing concentrations of NSC47924 or DMSO (■), as a vehicle control. Bound r37LRP was revealed by anti-His-HRP and OPD staining; the absorbance at 490 nm was measured. r37LRP binding to BSA-coated wells was subtracted to obtain specific binding. Values represent the mean ± SD of three experiments carried out in triplicate; (*, *P* < 0.05), as determined by the Student's *t* test. **C.** r37LRP was placed for 1 hour at 37°C on wells coated with 100 μg of YIGSR in the presence of increasing concentrations of NSC47924 or DMSO (■), as a vehicle control. Bound r37LRP was revealed by anti-His-HRP and OPD staining; the absorbance at 490 nm was measured. r37LRP binding to BSA-coated wells was subtracted to obtain specific binding. Values represent the mean ± SD of three experiments carried out in triplicate; (*, *P* < 0.05), as determined by the Student's *t* test. **D.** Predicted binding mode of NSC47924. The most populated and lowest energy pose is shown for NSC47924 docked into the 37LRP crystal structure (slate blue cartoon). NSC47924 is shown in stick, with carbons in green, nitrogens in blue, and oxygen in red. H-bonds interactions are shown with dashed black lines. The protein residues are shown in yellow stick, while the palindromic key residues are displayed in blue sticks. Human recombinant 37LRP binds LM and is inhibited by NSC47924; NSC47924 engages many contacts with residues of 37LRP peptide G.

In order to demonstrate r37LRP ability to bind LM, serial dilution of purified His-tagged r37LRP was incubated on wells pre-coated with LM, and binding was detected by anti-His HRP. As a control for binding specificity, r37LRP binding to BSA-coated wells was also evaluated in parallel and the OD readings subtracted. r37LRP specifically bound to LM, albeit with a lower efficiency than 67LR overexpressing cells, showing a K_d_ of 1.9 μM (Figure 5A), similar to that reported for a truncated form of 37LRP [2.3 μM; ref. 41].

Then, NSC47924 ability to inhibit r37LRP binding to LM was evaluated. NSC47924 inhibited r37LRP binding to LM, showing a IC_50_ of 58.9 μM and a K_i_ of 35.5 μM (not shown). Thus, NSC47924 is a specific inhibitor of 37LRP/67LR direct binding to LM (Figure 5B).

The precise contact site on LM for 67LR have not been mapped yet. However, it is believed that the interaction involves a specific peptide from the β1 chain of laminin (YIGSR) [42]; the binding activity of this synthetic peptide is increased by an amide group at the C-terminal [3]. Moreover, YIGSR is able to displace peptide G from LM, thus blocking the peptide G-mediated increase in LM degradation [18–19]. For these reasons, we investigated whether NSC47924 was able to compete for r37LRP binding to YIGSR. NSC47924 significantly inhibited r37LRP binding to YIGSR (Figure 5C), leading us to hypothesize that our target sequence, peptide G, could be effectively addressed by NSC47924.

Docking experiments were performed to gain insight into the possible mechanisms of interaction between NSC47924 and peptide G of 37LRP. As shown in Figure 5D, the naphthol ring of the ligand is sandwiched between two α-helices (αC and αE) of 37LRP and held in place by a H-bond formed between the hydroxyl group of NSC47924 and the G172 CO backbone. The naphthol moiety also engages a network of hydrophobic contacts with F90, W175, A86, G172, W176, A93, A179 residues. In addition, the pendant *p*-methoxy phenyl ring makes hydrophobic interactions with the palindromic residues W176 and L173. The aromatic side chain of W176 establishes an aromatic π-stacking interaction with the *p*-methoxy phenyl ring, contributing to further stabilize the complex. The latter residues have been shown to be critical for specific binding of LM to 37LRP [35]. Close inspection of the NSC47924/37LRP complex also shows that the bulky *p-*metoxy group of the ligand makes van der Waals contacts with the side chain of K17.

### Refinement of lead 67LR inhibitor NSC47924

Hierarchical screening is an efficient strategy that allows an initial broad search over a chemically and pharmacologically diverse set of compounds, followed by a focused search over a much larger database to find molecules related to potential lead compounds. A similarity search over the full NCI database with NSC47924 resulted in 108 compounds, which we docked to the putative cavity found in close proximity of 37LRP crystal structure and ranked according to predicted binding energy. The top 19 compounds based on a binding energy cutoff of −7 kcal/mol were selected for experimental testing (compounds 47–65 in Chart 2 of Supplementary Material: Refinement of lead 67LR inhibitor NSC47924).

**Figure 6:**
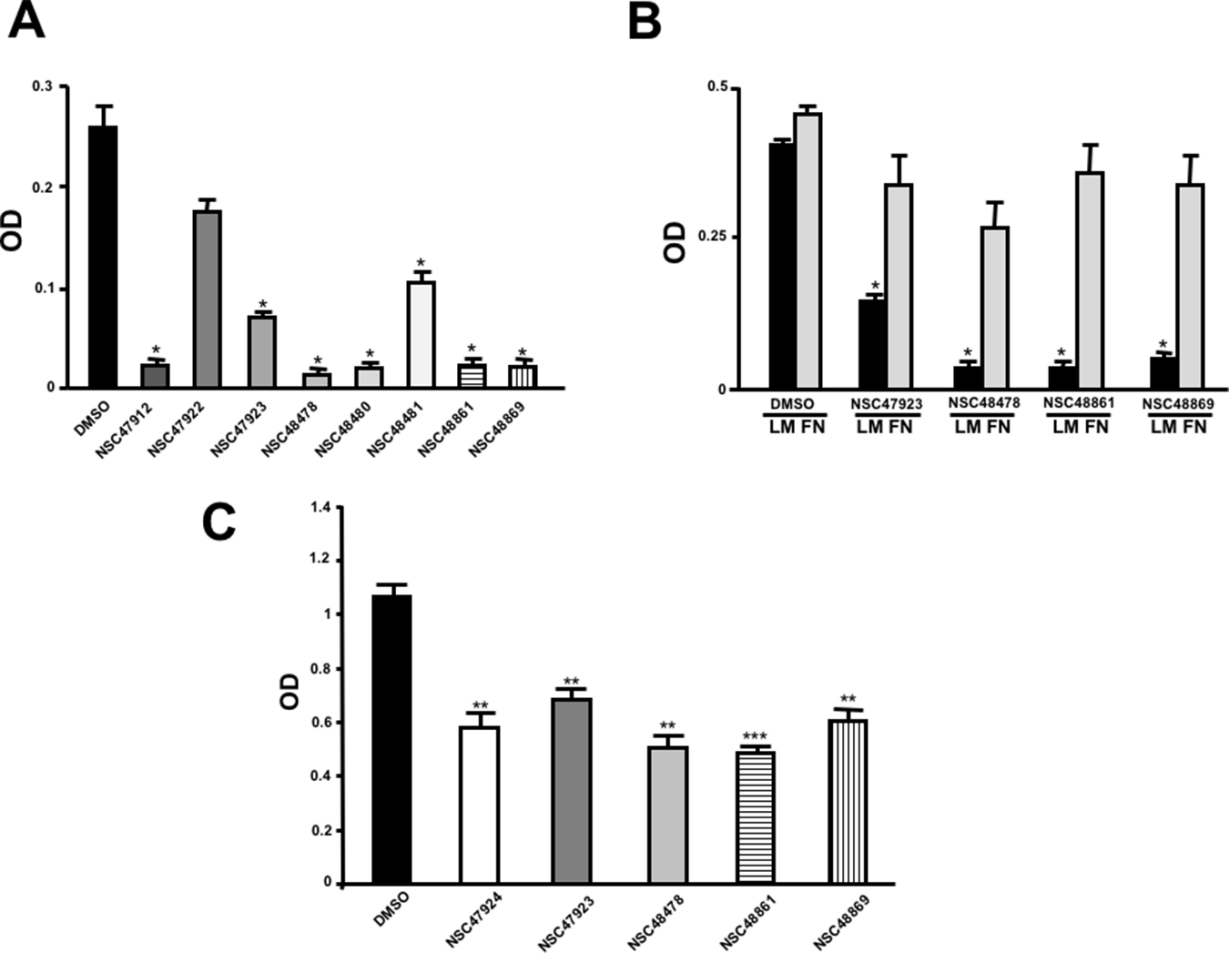
Refinement of lead 67LR inhibitor NSC47924 **A.** LR-293 cells were plated in LM-coated wells in the presence of the selected molecules, dissolved in DMSO at a concentration of 20 μM, DMSO was used as negative control (■). The attached cells were stained and the absorbance at 540 nm was measured. The values represent the mean ± SD of three experiments performed in triplicate. (*) *p* < 0.05, as determined by the Student's *t* test. Among the 19 small molecules tested, only eight compounds were able to inhibit LR-293 cell binding to LM and are shown in figure. **B.** LR-293 cell adhesion to LM (■) and FN (□), in the presence of 20 μM of inhibitory molecules or DMSO, as a negative control. The attached cells were stained and the absorbance at 540 nm was measured. The values represent the mean ± SD of three experiments performed in triplicate. (*) *p* < 0.05, as determined by the Student's *t* test. Only four compounds specifically decreased LR-293 cell binding to LM, without affecting cell adherence to FN and are shown in figure. **C.** r37LRP was placed for 1 hour at 37°C on LM-coated wells in the presence of NSC47924 and selected analogs at a concentration of 50 μM; DMSO (■), was used as a vehicle control. Bound r37LRP was revealed by anti-His-HRP and OPD staining; the absorbance at 490 nm was measured. r37LRP binding to BSA-coated wells was subtracted to obtain specific binding. Values represent the mean ± SD of three experiments carried out in triplicate; (*, *P* < 0.05; **, *P* < 0.01; ***, P < 0.001), as determined by the Student's *t* test.

The 19 top-ranked compounds identified in the similarity screen were tested at 20 μM concentration by *in vitro* cell adhesion assay to LM. Eight compounds were able to inhibit the adhesion of LR-293 cells to LM (Figure 6A), but only four of them, NSC47923 [1-(4-toluidinomethyl)-2-naphthol], NSC48478 [1-((4-chloroanilino)methyl)-2-naphthol], NSC48861 [1-((((2-hydroxy-1-naphthyl)methyl)-4-methylanilino)methyl)-2-naphthol] and NSC48869 [1-((((2-hydroxy-1-naphthyl)methyl)anilino)methyl)-2-naphthol] (Table 2) selectively inhibited LR-293 cell binding to LM without exerting a significant effect on cell binding to FN.

**Table 2:** Experimentally Determined IC_50_ Values and Properties Predicted by QikProp of the four NSC47924 analogs selectively inhibiting LM binding

Cpd	Structure	NCI number	MW^a^	QP log *P*^b^	QP log *S*^c^	QP *P*_Caco_^d^	QP log Khsa^e^	IC_50_ (μM)
**50**	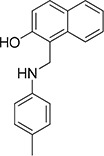	47923	263.3	4.0	−4.4	2999	0.453	1.9
**52**	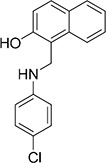	48478	283.8	4.5	−5.0	3491	0.510	1.8
**60**	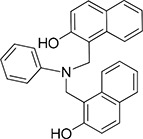	48869	405.5	6.0	−5.7	4484	1.032	4.0
**61**	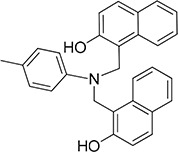	48861	419.5	6.3	−6.5	3026	1.236	3.4

a MW, range 95% of drugs (130/725).

b Predicted octanol/water log *P*, range 95% of drugs (–2/6.5).

c Predicted aqueous solubility, *S*, in mol/L, range 95% of drugs (–6.5/0.5).

d Predicted Caco-2 cell permeability in nm/s, range 95% of drugs (<25 poor, >500 great).

e Predicted binding to human serum albumin, range 95% of drugs (−1.5/1.5).

To also demonstrate the specificity for 67LR as a target, selected analogs were tested for their ability to inhibit the direct r37LRP binding to LM. NSC47923, NSC48478, NSC48861 and NSC48869 were active to the same extent as NSC47924, by *in vitro* binding assays (Figure 6C).

Interestingly, all active analogs showed IC_50_ values lower than NSC47924, when tested at decreasing concentrations on LR-293 cell adhesion assays (Table 2).

### Modes of binding for the top five inhibitors of 67LR

**Figure 7:**
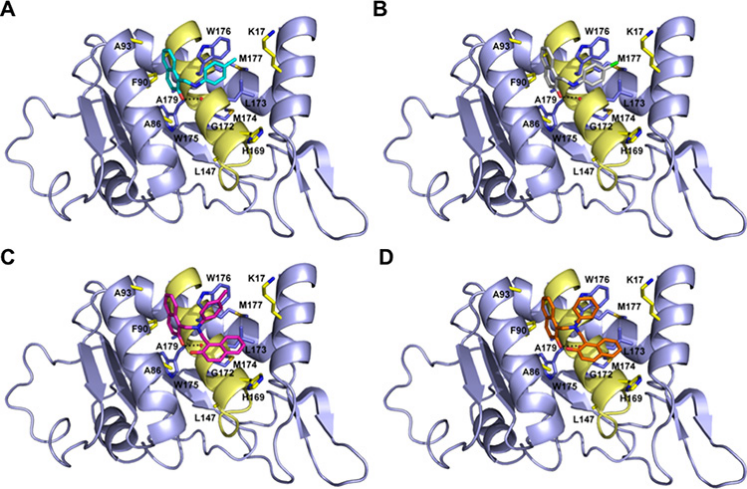
Predicted binding modes to 37LRP for NSC47923 (A. cyan), NSC48861 (B. white), NSC48869 (C. magenta) and NSC48478 (D. orange) Critical 37LRP binding residues are shown (yellow, carbon; red, oxygen; blue, nitrogen), while the palindromic key residues are displayed in blue sticks. H-bonds interactions are shown with dashed black lines.

The five micromolar drug-like inhibitors of 67LR that we have identified, all share a core 1-(anilinomethyl)-2-naphthol scaffold, which is predicted to bind in the deep putative 37LRP binding cleft (Figure 7). The core scaffold, including the H-bonding interaction with the carbonyl backbone of G172, conserves many protein-ligand interactions previously described for NSC47924 (Figure 5D). The naphthol moiety preserves hydrophobic interactions with F90, W175, A86, G172, W176, A93, and A179 residues, whereas the *p*-substituted phenyl ring maintains hydrophobic and aromatic π-stacking interactions with the palindromic residues W176 and L173. However, the differences of the *p*-substituents account for a large difference in activity. NSC47924 is 9.7-fold and 10.9-fold less active than NSC47923 and NSC48478, respectively. This suggests that the unfavourable steric interactions of the bulky methoxy group of NSC47924 and the K17 side chain would be removed by the less sterically demanding methyl group (NSC47923) and chlorine atom (NSC48478). The additional 2-naphtol ring in both NSC48861 and NSC48869 establishes favorable hydrophobic contacts with residues G172, L173 and H169, allowing the exploration of a rather lipophilic region of 67LR. One can speculate that specific ligand interactions with this region of the binding site could increase the inhibitory potency of these two compounds at 67LR.

### NSC47923, NSC48478, NSC48861 and NSC48869 inhibit tumor cell invasion

It has been widely reported that 67LR enhances cancer cell invasion and tumor metastasis by engaging LM and promoting ECM breakdown and the release of LM-derived motility fragments [18, 19].

Therefore, we sought to investigate whether selected compounds were able to inhibit the invasion through reconstituted basal membranes of highly invasive human fibrosarcoma HT1080 and human breast cancer MDAMB231 cell lines, constitutively expressing high 67LR levels [30, 43].

**Figure 8:**
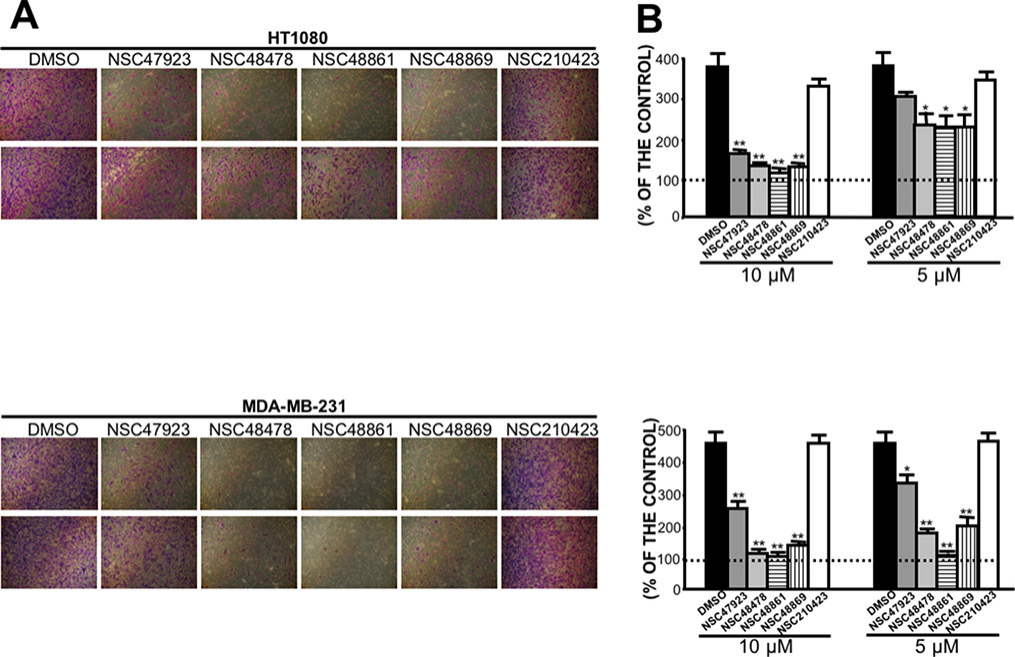
Selected close analogs of NSC47924 block very efficiently cancer cell invasion **A.** Highly invasive HT1080 fibrosarcoma cells (upper panel) and MDAMB231 human breast cancer cells (lower panel) were treated with DMSO, with 10 μM (first line) and 5 μM (second line) of compounds NSC47923, NSC48478, NSC48861, NSC48869 and NSC210423, as a negative control, and allowed to invade Matrigel, using as chemoattractant cell culture medium supplemented with 10% FBS. Invading cells on the lower side of the membranes were fixed, stained and photographed: a representative image is shown. **B.** Highly invasive HT1080 fibrosarcoma cells (upper panel) and MDAMB231 human breast cancer cells (lower panel) were treated with DMSO (■), with 10 μM and 5 μM of compounds NSC47923 (□), NSC48478 (□), NSC48861 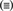, NSC48869 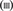, and NSC210423, as a negative control (□), and allowed to invade Matrigel, using as chemoattractant cell culture medium supplemented with 10% FBS. Invading cells on the lower side of the membrane were fixed, stained and counted and the results were expressed as a percentage of invasion in the absence of chemoattractant. The values are the mean ± SD of three experiments carried out in triplicate; (*, *P* < 0.05; **P < 0.01), as determined by the Student's *t* test. FBS-induced cancer cell invasiveness was significantly reduced by compounds NSC47923, NSC48478, NSC48861, NSC48869, at both concentrations tested.

All compounds strongly reduced Matrigel invasion, as compared with DMSO-treated cells (Figure 8A). Interestingly, selected compounds were still active at concentrations of 10 μM and 5 μM (Figure 8B), thus highlighting an increased *in vivo* activity, as compared to NSC47924 (Figure 4C), most likely due to their lower IC_50_ (Table 2). NSC210423, an inactive analog derived from the 19 top-ranked compounds identified in the similarity screen (Chart 2 of Supplementary Material), was added as a negative control and was ineffective in inhibiting Matrigel invasion, as expected (Figure 8A, 8B).

Therefore, four newly identified small-molecule inhibitors of 67LR are able to block cancer cell invasion by targeting the direct 67LR binding to LM, at low micromolar concentrations.

## DISCUSSION

Virtual screening (VS) has been successfully adopted as an attractive and complementary alternative to expedite and facilitate new lead compound identification for a specific biological target from large chemical libraries. Typically, VS encompasses a variety of computational techniques to prioritize compounds and it is fast and cost-effective in comparison to experimental high-throughput screening (HTS), thereby coming to the forefront in the modern drug discovery process [32]. There are two fundamental types of VS applications: structure-based virtual screening (SB-VS) and ligand-based virtual screening (LB-VS). SB-VS uses the knowledge of the three-dimensional structure of the target to select candidates most likely to interact with the binding site of the target [33], while LB-VS employs information of the known active compounds as a search query to retrieve the potential actives from large chemical libraries [44]. The main advantages of SB-VS methods compared to LB-VS methods are the structural novelty of the hits discovered (not based on pre-existing, known ligands) and the possibility to model the binding mode of potential ligands within the binding site.

Availability of large number of protein targets in cancer offers an excellent opportunity to carry out VS. In cancer research, successful VS strategies to identify novel hits have been reported by various groups [45]. However, identified hits cannot directly enter into clinical trials, but may serve as starting point for further optimization. Therefore, the VS route of cancer drug discovery provides an excellent opportunity to save time and money, bringing down the cost.

67 kDa laminin receptor (67LR) overexpression correlates with an enhanced invasive and metastatic potential in many human tumors [22–24] recommending the receptor as a new promising target for cancer drug discovery [25]. This is supported by *in vivo* studies showing that 67LR downregulation by antisense RNA reduces tumor cell proliferation and tumour formation [46]. Our group recently demonstrated that, upon LM binding, 67LR association with the anti-apoptotic protein PED/PEA-15 activates a signal transduction pathway, leading to cell proliferation and resistance to apoptosis [28]; indeed, siRNA mediated 67LR downregulation reduces cell viability by inducing apoptosis [27].

However, the main function of 67LR is to enhance tumor cell adhesion to the LM of basement membranes and cell migration, two crucial events in the metastasis cascade [20, 21]. Thus, inhibiting 67LR binding to LM could be a feasible approach to block metastatic cancer cell spread. Recently, several studies have shown that anti-37LRP/67LR specific antibodies significantly reduce the invasive potential of HT1080 fibrosarcoma, lung, cervical, colon, prostate, breast and oesophageal cancer cells [30, 43, 47], thus indicating that 67LR plays a critical role in tumor invasion and metastasis through its interaction with LM. Furthermore, 67LR interaction with LM might also be involved in angiogenesis [48] and recent findings suggest that the same anti-37LRP/67LR antibody is also able to block angiogenesis [29].

Thus, the main goal of our study was to identify small molecules able to inhibit 67LR interaction with LM. In our search for active compounds, we reviewed biological and biochemical data to identify a significant 67LR binding site for LM to target. We focused on the 37LRP-derived “peptide G” sequence (residues 161–180, IPCNNKGAHSVGLMWWMLAR) that binds LM with high affinity (K_d_ = 51.8 nM) [11, 15, 17], elutes 67LR from LM affinity chromatography columns [17], and mediates many functional effect linked to tumor progression, such as increased cell adhesion and migration to exposed ECM [18], increased tumor cell adhesion to endothelial cells and metastasis formation [15] and release of motility fragments from LM [19].

Interestingly, the mature 67LR originates from a ribosomal protein that acquired the LM binding function during evolution. Indeed, both human 37LRP and the p40 ribosomal protein are encoded by the same gene [9, 10]. Sequence conservation of 37LRP/p40 genes across species has been demonstrated, with evolution of the C-terminal tail, where is located the LM binding function, convergent with vertebrates [35]. Evolutionary studies also showed that the acquisition of LM-binding capability is linked to the palindromic sequence LMWWML contained within peptide G, which appeared during evolution concomitantly with LM or LM-like molecules. This consistent with the observation that two consecutive tryptophan residues are often associated with protein-protein interaction [49].

The fact that specific amino acid/s could be responsible for the functionality of the complex between 67LR and LM increased the possibility that a small molecule could exert a disrupting effect. Although an antibody and a peptide could also be able to target such interaction, we believe that a small molecule with the same activity would be preferable. Since disruption of LM/67LR interaction should block tumor cell invasion rather than cause cell death, to be an effective treatment, the drug would have to be used chronically in patients suspected to have overt disseminated or residual disease. A successful outcome implies prolonged treatments and thus the need for a drug with oral availability and high index of specificity for cancer cells. Because the library was selected on the basis of Lipinski's rule for drug-like properties [50], and because of the target, which is an interaction that takes place when 67LR is over-expressed, a condition of malignant tumors, those two requirement should be satisfied.

The crystal structure of 37LRP reveals that the surface loop of residues 187–205 completely covers peptide G, making it inaccessible. Thus, to expose the palindromic sequence, we truncated the surface loop of 37LRP and intercepted a hydrophobic cavity in close proximity of the palindromic residue W176. This putative cavity was chosen for *in silico* targeting in a SB-VS approach, which has already been used to successfully identify small molecule inhibitors of urokinase receptor (uPAR) [51], Cdc25B dual specificity phosphatases [52] and frataxin ubiquitination [53].

This approach led to the identification of a small molecule, NSC47924, with 67LR/LM disrupting activity. Since, small molecules have a higher likelihood for off-target effects (based on non-selectivity) in respect to protein-like biologicals [54], we demonstrated that NSC47924 reduced adhesion and migration to LM as well as Matrigel invasion of 67LR-overexpressing cells, without exerting any significant effect in vector-transfected cell, poorly expressing 67LR.

NSC47924 is expected to reduce cancer cell spread, rather than cause acute cancer cell death; however, we cannot exclude that NSC47924 can diffuse through the cell membrane, or be internalized upon 67LR binding, and thus exert also a role on cell proliferation and survival by targeting cytosolic, ribosomal and/or nuclear 37LRP.

Indeed, one of the most important advantages of small molecules, in respect to monoclonal antibodies, is cell membrane permeability. Moreover, the short half-life of small molecules may be of considerable clinical benefit in tailoring personalized target therapies in cancer, as it is emerging in rheumatology [55].

Hierarchical screening over the full NCI database with NSC47924, molecular docking studies and cell adhesion experiments identified four NSC47924 related compounds, NSC47923, NSC48478, NSC48861 and NSC48869, that showed increased 67LR inhibition ability, as compared to NSC47924.

In summary, we combined SB-VS experiments with *in vitro* assays and found five active molecules that were able to selectively disrupt the 67LR/LM activity in a timely and cost-effective fashion. In particular, we have identified a specific inhibitor of cell binding to LM, NSC47924, which is cell-permeable and selectively target 67LR-mediated cell adhesion, migration and invasion as well as four its close analogs (NSC47923, NSC48478, NSC48861 and NSC48869), even more active. This specificity, together with its simple chemical structure, make this compound class suitable for further development of novel therapeutics for the treatment of 67LR-dependent human malignancies.

## MATERIALS AND METHODS

### Computational chemistry

Molecular modeling and graphics manipulations were performed using Maestro (Maestro, version 9.9, Schrödinger, LLC) and Pymol [56] software packages running on a E4 Computer Engineering E1080 workstation provided of a Intel Core i7–930 Quad-Core processor.

### Protein preparation

The X-ray coordinates of the 37LRP fragment (PDB code 3BCH) [31] were extracted from the Protein Data Bank [57]. The structure was then prepared using the Protein Preparation Wizard of the Schrödinger graphical interface Maestro. X-ray water molecules and surface loop domain (residues 187–205) were removed during protein preparation, the last step of which was energy minimization of the entire structure. The minimization was terminated when the root mean square deviation (rmsd) of the heavy atoms in the energy minimized structure relative to the starting (X-ray) coordinates exceeded 0.3 Å.

### Ligand preparation and filtering

3D structures of NCI Diversity Set (1, 990 compounds) were extracted by the web site http://dtp.nci.nih.gov/branches/dscb/repo_open.html/ and prepared using LigPrep's ligand preparation protocol (LigPrep, version 3.1, Schrödinger, LLC). So, about 2, 560 structures including stereoisomers, tautomers, and ionization states were ready to be submitted to the subsequent docking runs.

### SB-VS protocol

The Glide v6.4 (Glide, version 6.4, Schrödinger, LLC) [37] VS application in Schrödinger Small-Molecule Drug Discovery Suite was used to screen the compound library using two levels of docking precision. In the first step, Glide was run in Standard Precision (SP) mode. The 10% of the top-scoring ligands (188 compounds) were kept and redocked using the Glide Extra Precision (XP) mode, which incorporates a more accurate, finer-grained docking algorithm, designed to eliminate false positives that survive the SP stage. After visual inspection of the top ranked compounds, 46 hits were chosen for biological evaluation. The grid for docking studies was chosen sufficiently large to enclose all residues involved in the palindromic sequence of peptide G (LMWWML) within a cubic box of dimensions 40 Å × 40 Å × 40 Å. The enclosing box was centered on the palindromic sequence setting the bounding box with the sizes of 14 Å × 14 Å × 14 Å. A van der Waals radius scaling factor of 0.80 for atoms with a partial atomic charge (absolute value) less than 0.15 was used in order to soften the potential for nonpolar parts of the receptor. Compounds identified by SB-VS were flexibly docked using the same protein grid prepared for SB-VS protocol. Ten poses were collected for each ligand and ranked according to predicted Glide XP score.

### Hierarchical similarity search

An online search utility provided by the NCI (http://129.43.27.140/ncidb2/) was used to search the entire NCI database for compounds similar to NSC 47924. Two methods were used to judge compound similarity: search on the basis of substructure by SMILES string (http://daylight.com) and/or similarity by Tanimoto coefficient, [58] with a cutoff of 0.85 [59]. From these searches, selected compound structures were docked to the putative LM binding pocket and ranked according to predicted Glide XP score. Compounds with the lowest scoring value were requested and assayed for effect on cell adhesion inhibition.

### Chemical inhibitors

All compounds identified by SB-VS were obtained from the NCI/DTP Open Chemical Repository (http://dtp.cancer.gov), dissolved in dimethyl sulfoxide (DMSO) and stored at −20°C, at a concentration of 0.01 M.

### Epitope tagging of the 37LRP for eukaryotic cell transfection

The 37LRP cDNA was fused at the carboxy terminus with a sequence coding for both a T7 tag and a 6xHis tag, by a two step procedure. First, the 37LRP coding sequence was PCR amplified from plasmid pPLR1.1 [8], kindly provided from Dr. M.E. Sobel, (Bethesda, MD), using oligonucleotides PR11 (GATCCCATTGATGTCCCCAGCCCTTGACGT) and PR12 (GATCCCATGGACCACTCAGTGGTGGCT) as 5′ and 3′ primers, respectively. The product was then cloned in frame into the Nco I site of the pTRcHisB vector (Invitrogen, Carlsbad, CA, USA), upstream the T7-tag and His-tag sequences. Second, the chimeric sequence was PCR amplified using oligonucleotides PR10 (GATCAAGCTTATGTCCGGAGCCCTTGACG) and PR5 (AGCTTCTAGATCAGAGCTCGGATCCTTATCGTC) as 5′ and 3′ primers, respectively. The product was cloned into the Hind III and Xba I sites of pcDNA3 eukaryotic vector (Invitrogen). The resulting plasmid was named pPLR2.5.

### Cell cultures and transfections

The human embryonic kidney cell line HEK-293 (ATCC Certified from LGC Standards, Milan, Italy) was grown in Dulbecco's modified Eagle's medium (DMEM) (GIBCO, Gaithersburg, MD, USA) supplemented with 10% fetal bovine serum (FBS).

5 × 10^6^ HEK-293 cells, cultured overnight in 100 mm tissue culture dishes, were stably transfected with 10 μg of pPLR2.5, or pcDNA3 and 60 μl of LipofectAMINE (Invitrogen) for 5 h at 37°C (5% CO2). Transfected cells, named LR-293 and V-293 respectively, were selected by Geneticin (GIBCO) at 1.5 mg/ml, pooled, and cultured in the presence of 0.5 mg/ml Geneticin.

67LR highly expressing HT1080 fibrosarcoma [30] and MDAMB231 breast cancer cell lines [43] were grown in DMEM supplemented with 10% FBS.

### Production of recombinant 37LRP and anti-67LR antibodies

A recombinant polypeptide was made in bacteria for production of anti-37LRP/67LR antibodies, as described [23].

### Flow cytometry

Flow cytometric analysis of cell surface molecules was performed as previously described [59].

### Western blot

Cells were lysed in PBS (0.08 M NaCl, 0.002 M KCl, 0.0115 M Na2HPO4, 0.002 M KH2PO4, pH 7.2) containing 1% Triton X-100, in the presence of a protease inhibitor cocktail containing AEBSF, Aprotinin, Bestatin, E-64, Leupeptin and Pepstatin A (Sigma-Aldrich, St. Louis, MO, USA) and a phosphatase inhibitor cocktail containing microcystin LR, cantharidin, and bromotetramisole (Sigma-Aldrich). Protein concentration of lysates was determined using a colorimetric assay (Biorad, Richmond, CA, USA). Equal amounts of protein were subjected to SDS-polyacrylamide gel electrophoresis, transferred to poly-vinyl difluoride (PVDF) filters (Millipore, Windsor, MA, USA), then subjected to Western blot with a polyclonal anti-37LRP/67LR antibody or with non-immune immunoglobulins (Igs) (Jackson ImmunoResearch, Suffolk, England), as decribed [23, 60].

### Cell adhesion assay

The adhesion assays were carried out on 96-well flat-bottomed plates (Nunc, Roskilde, Denmark) coated with 1 μg of laminin (LM) (Becton Dickinson Biosciences, Franklin Lakes, NJ, USA), fibronectin (FN) (Roche, Indianapolis, USA), vitronectin (VN) (Becton Dickinson Biosciences, Franklin Lakes, NJ, USA) or with 100 μl of heat-denatured 1% Bovine serum albumin (BSA) in PBS, as a negative control, as described [23, 28, 60, 61]. A binding curve and K_d_ were generated for compounds using GraphPad Prism software.

### Cell migration and invasion assays

Cell migration assays were performed in Boyden chambers using 8 μm pore size polyvinylpyrrolidone (PVPF)-free chemotaxis filters (Wathman Int., Kent, UK), coated with 50 μg/ml collagen, as an adhesion substrate. V-293 and LR-293 cells (2 × 10^5^) were plated in the upper chamber in DMEM 0.1% BSA containing the selected small molecules or DMSO, as a vehicle control. DMEM 0.1% BSA alone or containing 50 μg/ml LM was added in the lower chamber and the cells were allowed to migrate for 4 h at 37°C, 5% CO_2_.

For the invasion assay, filters were coated with 50 μg/ml Matrigel^TM^ (BD Biosciences, San Jose, CA) and incubated for 30 min at 37°C for gelling. 2 × 10^5^ cells, plated in the upper chamber in DMEM 0.1% BSA containing the selected small molecules or DMSO, were allowed to migrate toward DMEM medium supplemented with 10% FCS, or toward DMEM medium supplemented with 0.1% BSA, as a control, for 18 h at 37°C, 5% CO_2_.

At the end of both experiments, cells on the lower surface of the filter were fixed in ethanol, stained with hematoxylin, and counted at 200× magnification (10 random fields/filter). Cell migration and invasion were expressed as a percent increase over the control.

### Binding of soluble r37LRP to immobilized LM and YIGSR peptide

High binding plates with 96 flat-bottomed wells (Corning, Amsterdam, ND) were coated with 1 μg/well of LM diluted in PBS, or BSA as a negative control, and incubated at 4°C overnight. After a wash in PBS, residual binding sites were blocked for 1 h at 37°C with 200 μl of blocking buffer (2% FCS, 1 mg/ml BSA, in PBS). Wells were incubated with increasing concentration of r37LRP (diluted in PBS, 1 mg/ml BSA), which contained a 6 × His-tag, for 1 h at 37°C. Each well was washed three times with wash buffer (0.5% Tween in PBS). Penta-His HRP conjugate (1:500) (Qiagen) was added for 2 h at room temperature. After washing, substrate solution was added and absorbance was detected at 490 nm on an ELISA plate reader (Bio-Rad). Binding affinity was determined by subtracting background absorbance (BSA wells). A binding curve and K_d_ were generated for r37LRP using GraphPad Prism software. The K_d_ was calculated using a one-site binding hyperbola and the equation Y = B_max_ × X/(K_d_+X).

For inhibition experiments, wells precoated with 1 μg LM or 100 μg peptide YIGSR-amide (Polypeptide group, Strasburg) were incubated with 2 μg r37LRP, alone or in the presence of different concentrations of NSC47924. Washings, detection and analysis were performed as above described.

## SUPPLEMENTARY CHARTS


