# Supplementary material for: Discovery of new small molecules inhibiting 67 kDa laminin receptor interaction with laminin and cancer cell invasion
Source: Oncotarget. 2015 May 29;6(20):18116–33. doi: 10.18632/oncotarget.4016 (PMC4627239; doi:10.18632/oncotarget.4016)
Supplement: Supplementary file 1 [file oncotarget-06-18116-s001.pdf]

SUPPLEMENTARY CHARTS

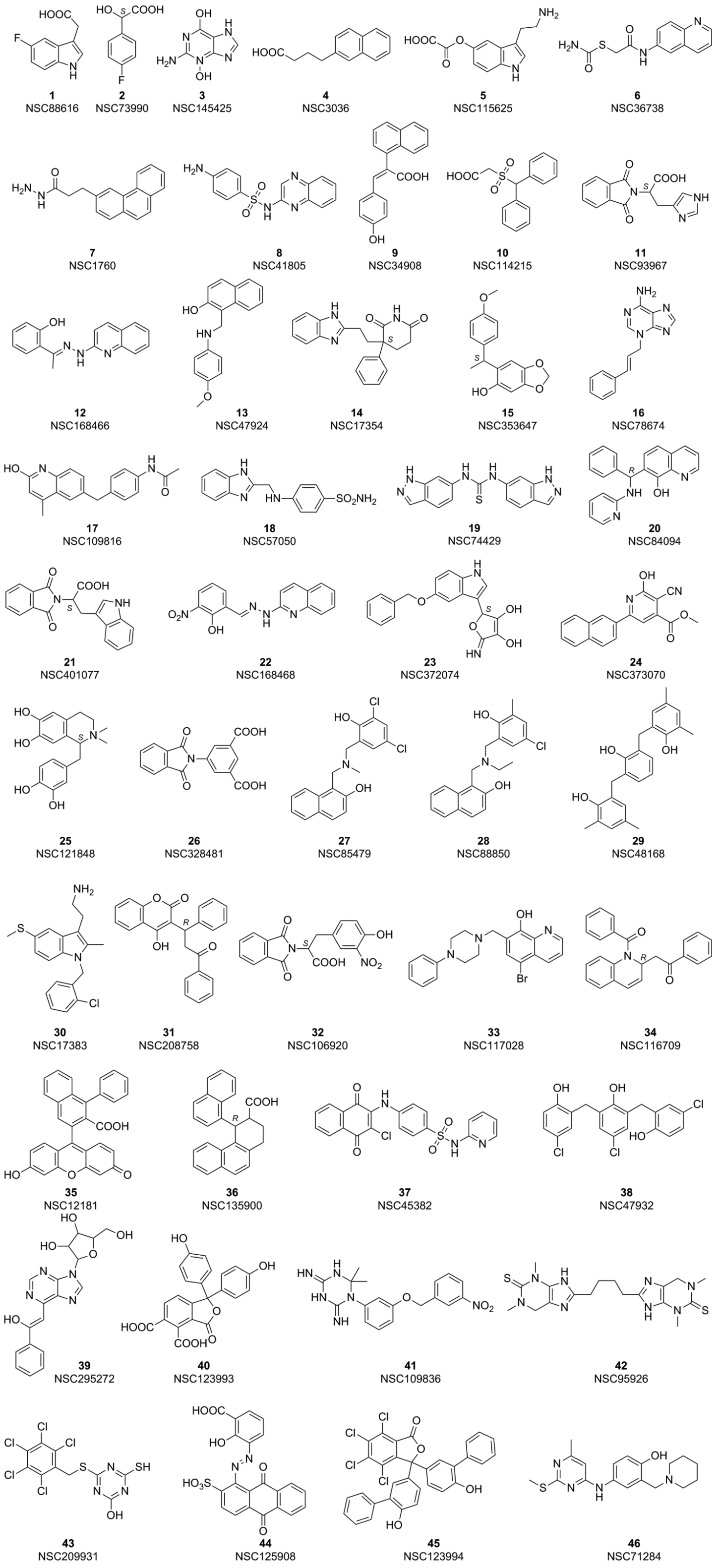

Supplementary Chart 1: SB-VS selected small molecules directed to the peptide G cavity of 67LR.

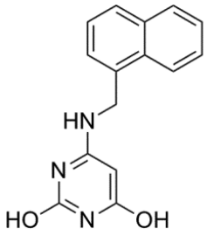

47
NSC210423

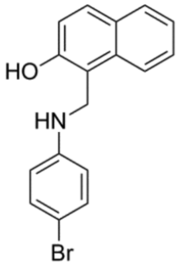

48
NSC47912

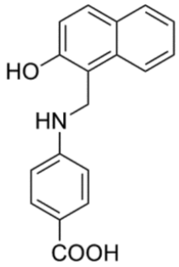

49
NSC47922

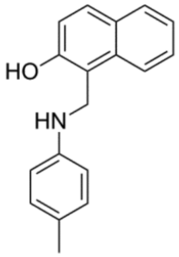

50
NSC47923

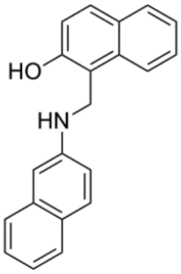

51
NSC47936

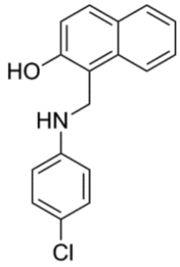

52
NSC48478

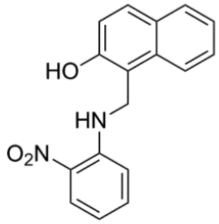

53
NSC48480

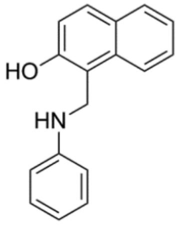

54
NSC48481

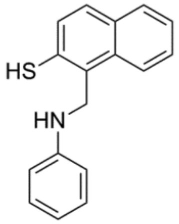

55
NSC89505

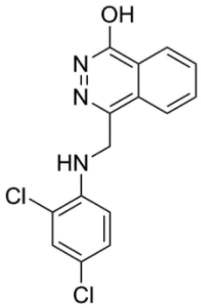

56
NSC646680

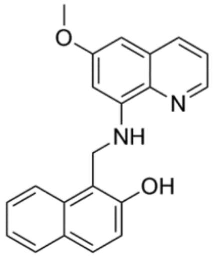

57
NSC130802

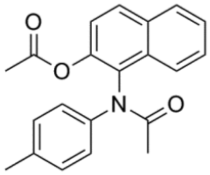

58
NSC47940

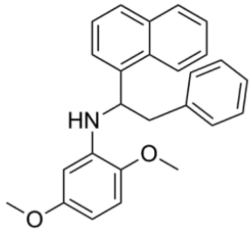

59
NSC403533

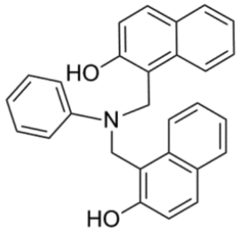

60
NSC48869

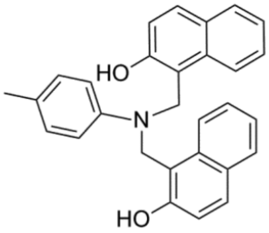

61
NSC48861

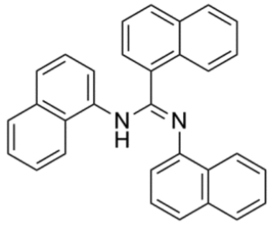

62
NSC44674

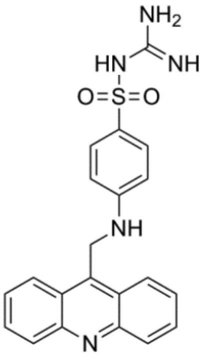

63
NSC348894

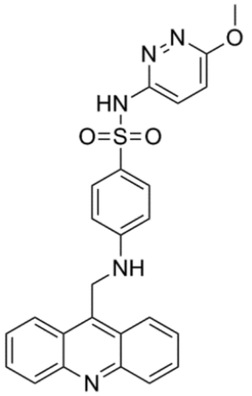

64
NSC348896

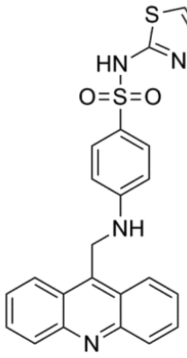

65
NSC353717

Supplementary Chart 2: Refinement of lead 67LR inhibitor NSC47924.
